# Analysis of Plant Physiological Parameters and Gene Transcriptional Changes Under the Influence of Humic Acid and Humic Acid-Amino Acid Combinations in Maize

**DOI:** 10.3390/ijms252413280

**Published:** 2024-12-11

**Authors:** Kincső Decsi, Mostafa Ahmed, Roquia Rizk, Donia Abdul-Hamid, Zoltán Tóth

**Affiliations:** 1 Institute of Agronomy, Hungarian University of Agriculture and Life Sciences, Georgikon Campus, 8360 Keszthely, Hungary; szaszkone.decsi.eva.kincso@uni-mate.hu (K.D.); roquiaibrahim@gmail.com (R.R.); toth.zoltan@uni-mate.hu (Z.T.); 2 Festetics Doctoral School, Institute of Agronomy, Hungarian University of Agriculture and Life Sciences, Georgikon Campus, 8360 Keszthely, Hungary; 3 Department of Agricultural Biochemistry, Faculty of Agriculture, Cairo University, Giza 12613, Egypt; 4 Heavy Metals Department, Central Laboratory for The Analysis of Pesticides and Heavy Metals in Food (QCAP), Dokki, Cairo 12311, Egypt; donia.atalah11@gmail.com

**Keywords:** bioactive humic acid and amino acid compounds, yield parameters, content characteristics, photosynthesis, carbon fixation, cellular respiration, oxidative phosphorylation, priming, mRNA sequencing, next-generation sequencing (NGS)

## Abstract

The study investigated the application of humic acids (HAs) and a combination of humic acids and amino acids (HA+AA) in maize under field conditions. Based on preliminary data in the literature, the aim was to investigate the effects of the two plant conditioning compounds on plant physiological parameters. In addition to measuring plant physiological parameters in the field, a complete transcriptome analysis was performed to determine exactly which genes were expressed after the treatments and in which physiological processes they play a role. Maize plants showed significant positive yield changes after two priming treatments. Genome-wide transcriptomic analysis revealed the activation of photosynthetic and cellular respiration processes, as well as protein synthesis pathways, which explains the increased yield even under extreme precipitation conditions. The results show that the HA treatment helped in water management and increased the chlorophyll content, while the HA+AA treatment led to higher protein and dry matter contents. The post-harvest tests also show that the HA+AA treatment resulted in the highest yield parameters. Functional annotation of the maize super transcriptome revealed genes related to translation processes, photosynthesis, and cellular respiration. The combined pathway analysis showed that the HA and combined treatments activated genes related to photosynthesis, carbon fixation, and cellular respiration, providing valuable in-depth insight into the usefulness of the HA and HA+AA treatments in priming. Based on the studies, we believe that the use of natural-based humic acid plant conditioners may provide a beneficial opportunity to promote renewable, regenerative agriculture.

## 1. Introduction

In the process known as humification (i.e., humus formation), plant and animal-origin substances undergo physical, chemical, enzymatic, and microbiological transformations to form humic substances (HS). Easily decomposable organic substances, under optimal conditions, quickly mineralize and turn into inorganic substances. Nitrogen-containing substances combine with a significant portion of the difficult-to-decompose compounds, transforming them into high-molecular, dark-colored, and relatively stable new compounds, known as humic substances, through chemical polymerization. Based on their solubility, humic substances can be classified into the following three groups: humic acids (HAs), fulvic acids (FAs), and humins [1].

Both the primary assimilative (e.g., photosynthesis, nucleic acid, and protein synthesis), dissimilative (e.g., cellular respiration), and also secondary metabolic processes are greatly affected by humic and fulvic acid content solutions [2,3,4,5]. Photosynthesis is one of the assimilative processes, which largely determines the amount of plant production. The chlorophyll-a and chlorophyll-b contents of the leaves of plants treated with humic acid are significantly higher than those of untreated plants. The relatively higher chlorophyll content causes the light phase of photosynthesis to work more effectively, thereby increasing the net photosynthesis rate [6,7,8,9].

The effects of leaf humic acid treatment on chrysanthemums were investigated in [9], highlighting the changes in photosynthesis and chloroplast ultrastructure. The results show that the humic acid treatment increased the chrysanthemum’s net photosynthesis rate and chlorophyll content and improved the structure of the chloroplast ultrastructure. However, humic and fulvic acid treatment can only be effective with an adequate microelement supply. The central core of chlorophyll molecules is magnesium, and the photochemically active centers that play an important role in binding light contain iron, sulfur, manganese, and calcium. After the absorption of light, some compounds included in the transformation processes taking place in the plant (e.g., cytochromes, plastocyanin, and ferredoxin) also contain iron, sulfur, and copper. The energy generated at the end of the light period is bound in ATP molecules in the plants, compounds of which require a significant amount of phosphorus [10]. The conversion of CO_2_ into organic matter takes place in the enzymatic stage [11]. The enzymes produced in this stage—in both C3 and C4 plants—contain magnesium. Chen et al. (2022) [12] grew maize plants in pots at two HA supply levels. The results show that the availability of phosphorus, potassium, iron, and magnesium in the soil improved and the activity of Rubisco and ATP synthase enzymes increased.

The operation of the stoma, which plays a role in photosynthesis and evaporation, can also be beneficially influenced by humic substances. The degree of stomatal resistance improves, meaning that the plant can reduce water loss through evaporation as the stomatal gaps narrow [13]. The intercellular carbon dioxide concentration decreases in parallel; thus, the plant can bind additional CO_2_ [14,15], and, ultimately, more organic matter is created, less water evaporates, and the operation of the plant becomes more economical (production of more organic matter with more economical water use).

Tejada et al. (2003) [16] applied foliar fertilization to asparagus plants using humic acid preparation. It was proven that the total and soluble carbohydrate contents of the plant’s organs increased due to the treatments. Humic substances also beneficially influence nucleic acid biosynthesis by promoting the formation of specific carbohydrate molecules (ribose and heptulose), which are essential in nucleotide biosynthesis. In addition, an increase in the level of another important component of nucleic acids, adenine, as well as adenosine derivatives, ribonic acid, and citric acid, was also demonstrated in the lettuce experiment [17]. Humic acid treatment also induces protein synthesis, and the amino acid content and the soluble protein content increase as a result [18]; in addition, the cellular metabolism improves, and the intensity of the lipid and amino acid metabolism increases [19].

The structure of humic substances practically acts as electron and oxygen-transporting molecules in biological systems. This ability allows them to participate in cellular respiration processes as catalysts. Accelerating these processes will indirectly increase the energy production of the citrate cycle. The citrate cycle is part of the metabolic pathway that converts carbohydrates, fats, and proteins into CO_2_ and water, producing cell energy.

In an experiment by Wang et al. (2023), in maize, it was shown that HA treatments beneficially affected several critical biochemical pathways, including the tricarboxylic acid (TCA) cycle. Several enzymes have been identified that are involved in different plant responses to HS, such as tricarboxylic acid cycle enzymes [20] and glycolytic enzymes [21].

Increased amino acid and lipid biosynthesis attributable to the beneficial effect of humic substances also contributes to tissue regeneration. Because of this, people often combine humic acid compounds with amino acid (AA) components, anticipating a synergistic effect. Researchers began investigating the potential of combining HS- and AA-containing compounds as early as the 1970s. Researchers discovered that while the two groups of compounds do not interact, the HS components do not hinder the degradation and unfolding of AAs [22]. Researchers also established that HSs contain AA components, including basic amino acids like arginine, histidine, and lysine, as well as acidic amino acids like aspartic acid and glutamic acid. According to their data, the amount of basic amino acids in HAs can increase as the humification level increases. This means that the makeup of HA fractions (and, by extension, their effects) are not always the same [23].

A combination of HAs and AAs was applied by El-Ghamry et al. (2009) [24] on fava beans. The combined active ingredients had a positive effect on plant growth (plant height, number of branches and leaves, number of pods per plant, and thousand-seed weight), chemical composition (N, P, and K in seeds and straw), chlorophyll content, and chocolate spot and rust diseases. In Egypt, researchers conducted two-year studies on three bread wheat varieties, utilizing HAs and a combination of HAs and AAs. Foliar spraying with a mixture of HAs and AAs resulted in a significant increase in crop yields. Compared to the control treatment, it increased grain and straw yield, protein, and carbohydrate content in both seasons. According to the same research group, adding the humin and amino acid mixture made the plant much taller, the stems wider, the leaves more considerable, the plant’s dry weight higher, and it had more chlorophyll-a, chlorophyll-b, carotenoid pigments, as well as proline. In addition, a higher total phenolic content was typical in the treated cereal plants [25].

According to Kenawy et al. (2017) [26], the combined treatment with HAs and AAs significantly increased the protein and proline contents, and the treatment containing high levels of AA also had the highest total chlorophyll content in the leaves. In experiments conducted on lettuce, Silva et al. (2019) [27] showed that using HAs alone or in combination with AA helps improve the morphology and quality of lettuce plants. Sadeghi Ghahnasir et al. (2023) [28] investigated the effect of applying humic acid and amino acids on tomatoes’ quantitative and qualitative characteristics. According to their results, foliar application of organic compounds improved the tomato yield, fruit components, and fruit quality. Similarly, positive results were reported following combined treatments in sweet corn [29], paspalum [30], mung bean [31], pomegranate [32], and anise [33].

However, the research results are not precise, as some studies reported that after the application of HA, no significant effect was observed either on crop growth or on the activity of soil life [6,34,35,36]. According to Karakurt et al. (2009) [37] in the paprika experiment, the application of HAs did not significantly affect the concentration of chlorophyll-a. Shehata (2011) observed the limited growth effects of foliar fertilizer containing humic acid and/or amino acids on strawberries [38].

During experiments by El-Bassiouny et al. (2014) [35], there were no significant differences in the wheat stem height, spike length, or yield. In a hydroponic study, HA treatment significantly boosted the activity of enzymes responsible for the reduction and assimilation of N in maize, yet this was negatively correlated with the protein content of maize leaves [39]. Abdel-Wahab and Shehata (2019) treated peppers with combinations of solutions containing HAs and AAs [40]. According to their findings, there were no significant differences compared to the control, except for the HA+AA treatment, which caused a significant reduction in fruit length compared to the control.

The application of HAs to the soil significantly increased the protein content of peanuts in the first growing year, but it had no significant effect on the protein content in the other 2 years of the experiment [41]. In another experiment, foliar application of HAs had no significant effect on the protein content of millet in field trials [42]. De Hita et al. (2020) [43] found that applying HA-containing solutions to leaves did not result in a short-term increase in abscisic acid’s root concentration or the H^+^-ATPase activity of the root plasma membrane.

Similarly, in a field experiment in Iran, treatments did not significantly increase the protein and gluten contents of wheat following HA foliar application [44]. Cha et al. (2020) [45] performed a transcriptomic analysis on the Arabidopsis plant and found that HA treatment downregulated most genes related to secondary metabolic pathways. Najafi et al. (2022) [46] treated cucumber plants under drought-stress conditions with a combined preparation of HAs and AAs, but the treatment proved ineffective. Sadeghi Ghahnasir et al. (2023) applied several treatments of plant conditioners containing HAs and/or AAs to tomato plants [28]. The results show that the plants’ highest fresh and dry weights were observed in control, water-treated plants.

Sible et al. (2021) stated that the application of HAs can also produce inconsistent yield results, probably due to the different biological origins of HAs [47]. Hose et al. (2000) found that a high concentration of HS in the plant reduces the hydraulic conductivity [48], thus inhibiting both the uptake of water and nutrients by the roots and the shoot growth. This can be attributed to the accumulation of HAs in the cell wall pores on the root surface [49]. We conducted a field experiment on maize plants with pure humic acid and humic acid combined with amino acid priming treatments to clarify the conflicting research results. We examined the plant’s physiological parameters and analyzed the mRNA profile changes due to the treatments using bioinformatics transcriptome analysis. Our goal was to more precisely identify the biochemical metabolic processes activated by the treatments and identify overexpressed genes to obtain more profound analytical results than the information available on the effect of HA and HA+AA on plant physiological processes. In addition, our goal was to confirm or reject the contradictory field measurement results at the gene level using modern transcriptomics tools.

## 2. Results

### 2.1. Weather Conditions During the Growing Season

**Figure 1:**
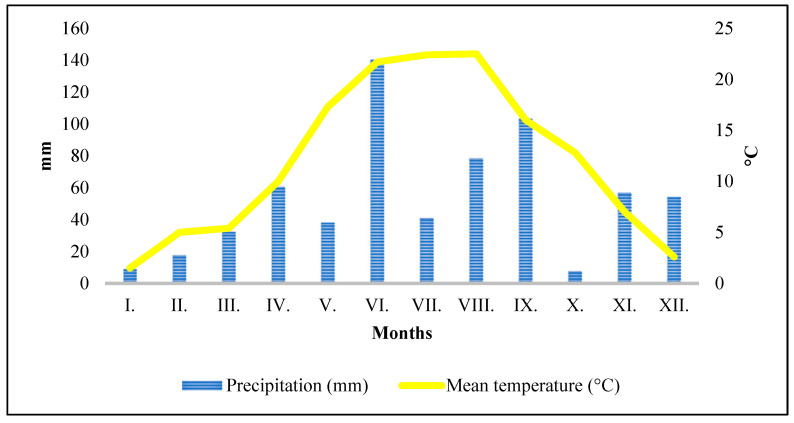
Weather trends in the treated area in 2022.

The long-term average annual temperature in Hungary’s plains ranges from 10.5 to 11.5 °C. Keszthely recorded an average of 12 °C in 2022. In the 2022 year, the monthly average was 21.7 °C in June and 22.4 °C in July, not exceeding the long-term average. The amount of rain in 2022, 639.8 mm, was nearly identical to Hungary’s typical annual precipitation of 600–700 mm, but the distribution was quite extreme. The long-term average rainfall in this area typically surpasses 70–70 mm during the treatment months, as shown in Figure 1. However, in 2022, the high precipitation in June (140.6 mm) and the exceptionally low water in July (41 mm) significantly hindered maize cultivation.

### 2.2. Water Saturation Tests

**Figure 2:**
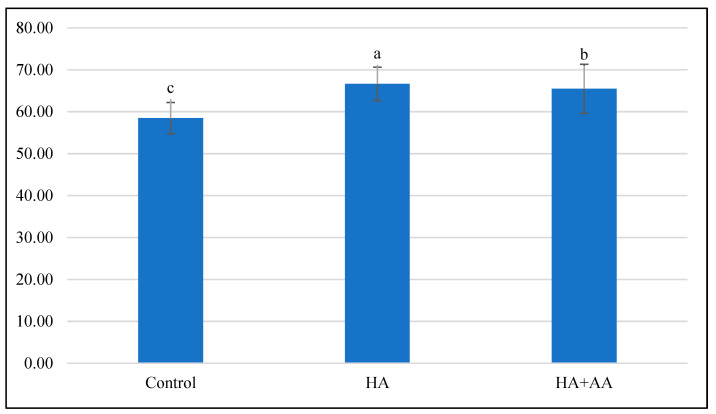
Current saturation deficit after the first treatment. The small letters above the bars (a, b, and c) indicate a statistically significant difference between variables. If two variables have different letters, they are significantly different. The level of significance was set at *p* < 0.05, with a confidence level of 95%.

**Figure 3:**
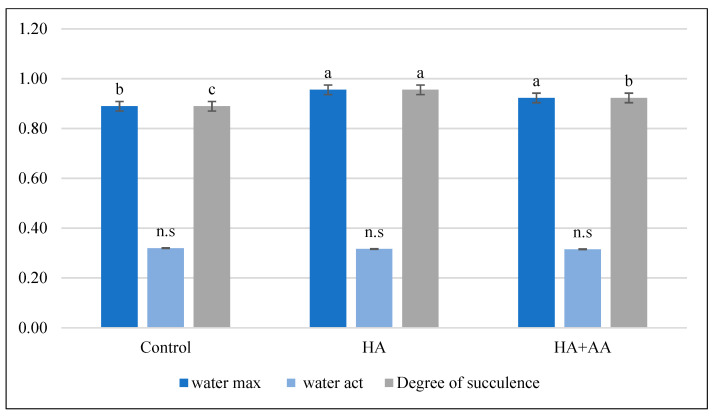
Water saturation values after the second treatment. The small letters above the bars (a, b, and c) indicate a statistically significant difference between variables. If two variables have different letters, they are significantly different. n.s. indicates a non-significant difference between variables. The level of significance was set at *p* < 0.05, with a confidence level of 95%.

The water saturation deficit calculations show that the treatments did not significantly affect the plants’ water management parameters. Only the HA treatment helped during the first treatment (Figure 2), but the HA+AA combined treatment was ineffective in this respect. The second priming did not significantly affect the plant’s water balance parameters (Figure 3). The second priming did not significantly affect the plant’s water balance parameters (Figure 3).

### 2.3. SPAD Measurements

**Figure 4:**
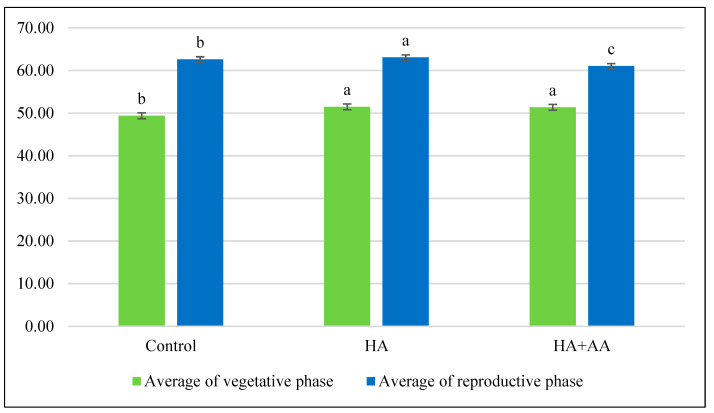
Chlorophyll content after the treatments. The small letters above the bars (a, b, and c) indicate statistically significant differences between variables. If two variables have different letters, they are significantly different. The level of significance was set at *p* < 0.05, with a confidence level of 95%. In this statistical analysis, the treatments were considered factors that could affect the chlorophyll content. The null hypothesis is that there was no significant difference between treatments.

In the case of chlorophyll content, the graph describes that there was a significant difference between the vegetative and reproductive phases (Figure 4). In general, the chlorophyll content was higher in the reproductive phase than in the vegetative phase. In addition, there was a significant difference between the treatments. In the HA treatments, a significantly higher chlorophyll content can be detected than in the combined treatment and in the untreated plots.

### 2.4. NIR Spectrometer Analysis

Regarding the NIR leaf results, the HA+AA treatment led to significant increases in the protein content (4.25), dry matter (23.81), ash (0.959), and acid detergent fiber (ADF; 4.92 compared with 4.94 with the HA treatment), and in the neutral detergent fiber (NDF), it was 13.72. All treatments led to results significantly higher than the control (Table 1).

In the NIR post-harvest measurements of grains, the starch concentration was significantly higher (59.34) with the HA treatment (Table 2).

### 2.5. Post-Harvest Tests

According to the yield parameters, the data in Table 3 show that the HA+AA treatment led to the highest yield parameters, except for the embedding parameter. HA+AA led to significantly higher values for weight (220.61 g), length (19.545 cm), diameter (2.16 mm), serial number per seed (36.2), number of maize kernels (428.3), cob weight (40.08 g), and grain weight (189.52 g). There is no significant difference between the number of lines between treatments (Table 3).

### 2.6. Transcriptomic Analyses

**Figure 5:**
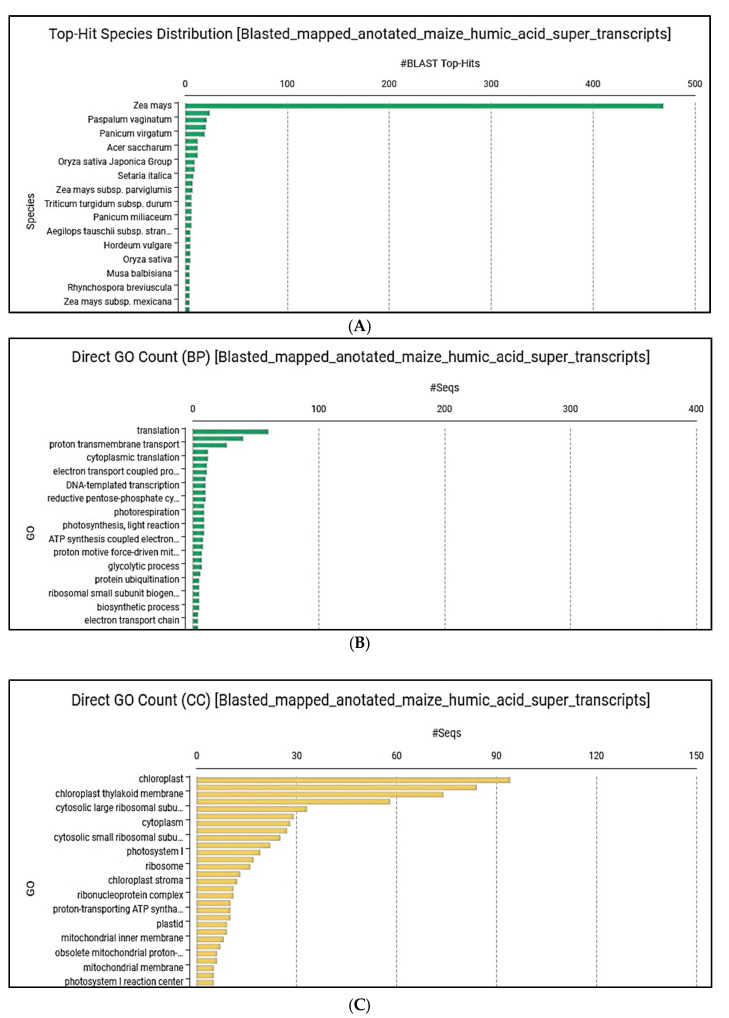

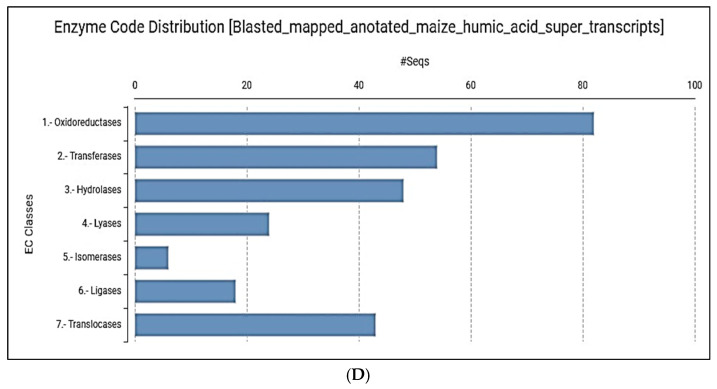
(**A**) Origin of transcripts; (**B**) biochemical processes involved in the effects of the treatments; (**C**) cell organelles involved in the effects of the treatments; (**D**) enzyme groups activated as a result of the treatments.

The entire super transcript (TSA) was blasted, mapped, and annotated, from which it was determined that a significant part of the contigs came from maize (*Zea mays* L.) (Figure 5A). Many contigs are genes involved in translational processes (e.g., enzyme formation), and, in addition, their high rate of participation in certain subprocesses of photosynthesis and cellular respiration is also clearly visible (Figure 5B). The distribution of the activated genes in cell organs also predicts a clear change in photosynthesis (Figure 5C).

In terms of enzymes, the high number of transcripts is significant in the case of oxidoreductases, which also confirms the activation of redox reactions (Figure 5D).

The count table was generated using TSA. The number of multiple aligned reads was 9206152 in the control, 8746638 with HA, and 10442557 with the HA+AA treatment. The count table yielded 1731 longer contigs (Supplementary Files, worksheet 1).

Using the count table, we conducted pairwise differential expression analyses between the control and HAs and the control and HA+AA treatments. The control vs. comparison with the HA treatments resulted in 633 differentially expressed sequences (probability > 0.9) from the 1731 examined contigs, of which 327 up- and 306 downregulated sequences were obtained (Supplementary Files, worksheet 2). For the comparative analysis of the HA+AA treatments with the control, we obtained 902 DEGs out of 1731 contigs (probability > 0.9), of which 407 and 495 were up- and downregulated (Supplementary Files, worksheet 3).

As a result of the gene enrichment analyses, 94 over- and 88 underrepresented genes were obtained from the control vs. HA treatment, while 148 over- and 121 underrepresented genes were obtained from the control vs. HA+AA treatment. During both comparisons, we identified individual genes, determined their GO terms, and performed their functional annotation. Among the genes activated as a result of humic acid treatments, 17 are related to certain partial processes of photosynthesis, while another 6 occur in defense responses to cellular respiration and 6 in ribosomal processes (Supplementary Files, worksheet 4). Among the 148 genes activated with the HA+AA treatments, 22 are involved in photosynthesis, 17 are involved in cellular respiration, and 7 in ribosomal processes (Supplementary Files, worksheet 5).

**Figure 6:**
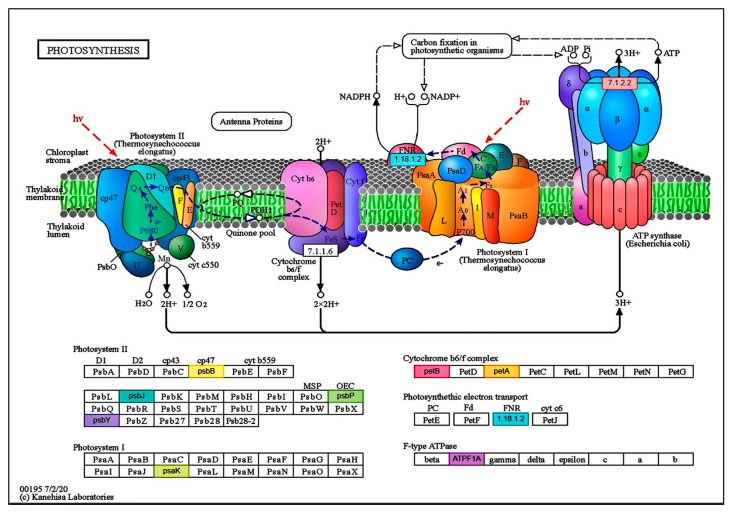
Genes activated by the HA treatments in photosynthesis. The figure was generated using the Combined Pathway Analysis submenu of the OmicsBox software https://www.biobam.com/omicsbox/ (accessed on 5 August 2024), and it is presented in its original form. The image visualizes the relationships among the identified genes that are statistical significant between the treatments. The abbreviations represent all genes and enzymes (EC code classification name) involved in the functioning of the given biochemical pathway, of which those marked in color underwent a significant change due to the treatment. A detailed analysis of the latter (colored) can be found in the text.

Analyzing the obtained results further, we examined the role of genes modulated by the treatments in the individual biochemical pathways. During the combined pathway analysis, we ran our identified sequences in the KEGG and Plant Reactome databases. Among the genes activated following the humic acid treatment, 47-47 genes activated by the HA treatments in photosynthesis were linked to different biochemical pathways in both databases (Supplementary Files, worksheet 6). Most of the genes are involved in assimilative processes, including photosynthesis (Figure 6).

In the light phase of photosynthesis, one gene in photosystem II and three genes in photosystem I were induced following the HA treatment. In addition, four additional genes were moved in the electron transport chain and ATPase enzyme activity. This pathway activates nine genes during the carbon-fixation step (Supplementary Files, worksheet 7). Furthermore, dissimilation processes induce certain genes. HAs activated four genes involved in glycolysis/gluconeogenesis processes (Supplementary Files, worksheet 8). The oxidative phosphorylation step, in which cellular respiration mostly produces ATP, exhibits an overrepresentation of genes (one NADPH dehydrogenase, two ATPases, and three additional genes in the electron-transport chain) (Supplementary Files, worksheet 9). In addition to these, we found another 3-3-induced gene in the large and small subunits of ribosomes (Supplementary Files, worksheet 10).

**Figure 7:**
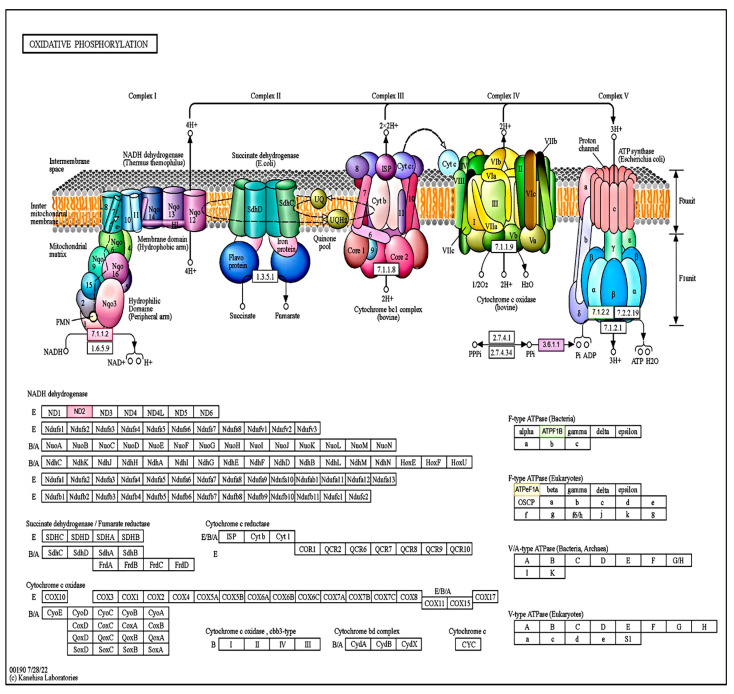
Genes activated by the HA+AA treatments in oxidative phosphorylation. The figure was generated using the Combined Pathway Analysis submenu of the OmicsBox software https://www.biobam.com/omicsbox/ (accessed on 5 August 2024), and is presented in its original form. The image visualizes the relationships between the identified genes that are statistically significant between the treatments. The abbreviations represent all genes and enzymes (EC code classification name) involved in the functioning of the given biochemical pathway, of which those marked in color underwent a significant change due to the treatment. A detailed analysis of the latter (i.e., colored) can be found in the text.

Among the genes activated by the treatments containing humic acid combined with amino acids, 52 in the KEGG database and 26 in the Plant Reactome database were connected to different biochemical pathways (Supplementary Files, worksheet 11). The majority of the overrepresented genes in these samples have an impact on certain parts aspects of photosynthesis (Supplementary Files, worksheet 12). These include the light phase (four genes in photosystem II, one gene in photosystem I, two genes in the cytochrome complex, seven genes in photosynthetic electron transport, and another gene that increases ATPase activity) and carbon fixation (seven genes) (Supplementary Files, worksheet 13). Furthermore, the combined treatment induced 17 genes during oxidative phosphorylation, a physiological process that responded with remarkable activity (Figure 7).

In addition to the small subunit, the large subunit of ribosomes also showed great activity. Compared to the control, we observed changes in six genes (Supplementary Files, worksheet 14).

Only one gene increased in the small subunit. The combined treatments also initiated new biochemical pathways, including purine metabolism (Supplementary Files, worksheet 15) and thiamine metabolism (Supplementary Files, worksheet 16), activating five genes. As a result of the humic acid treatments, the biochemical processes cysteine and methionine metabolism, fructose, mannose, galactose metabolism, linoleic acid metabolism, ascorbate, and aldarate metabolism, glyoxylate and dicarboxylate metabolism, biosynthesis of various antibiotics, biosynthesis of various plant secondary metabolites, glutathione metabolism, methane metabolism, cyano-amino acid metabolism, thermogenesis, inositol phosphate metabolism, porphyrin metabolism, amino sugar and nucleotide sugar metabolism, starch and sucrose metabolism, sulfur metabolism, and degradation of flavonoids were overrepresented and downregulated based on the KEGG analyses (Supplementary Files, worksheet 17).

Similarly, the HA and AA combined treatment led to the overrepresentation and downregulation of certain genes. These genes are involved in biochemical processes, as listed in Supplementary Files, worksheet 18. Note that the metabolism of methane and pyruvate downregulates 4-4 genes each. Both treatments organized the upregulated genes into larger clusters, which could significantly impact the functioning of specific biochemical processes such as photosynthesis (15 genes) and oxidative phosphorylation (17 genes). In comparison, the downregulated genes only repressed a maximum of 1-4 genes per biochemical cycle.

In summary, the HA treatments primarily induced photosynthesis and cellular respiration processes (17 and 10 genes), whereas the HA+AA combined treatment enhanced this effect by modulating additional genes (photosynthesis: 22 genes; cellular respiration: 17 genes). In addition, both treatments positively affect the activation of ribosomal processes (six and seven genes, respectively).

## 3. Discussion

We applied the humic acid and HA+AA combined treatments to maize plants to gain a deeper understanding of the effects of the two components and a genome-wide analysis of their effects at the transcription level. During our tests, we established that treatments containing pure HAs promoted the functioning of specific partial processes of photosynthesis. As a result, the activation of the PsbP protein was observed in the OEC complex of photosystem II (PSII), which plays a role in optimizing oxygen emission [50], and the induction of PsaF, PsaH, and PsaL proteins was also observed in photosystem I (PSI). The PsaF protein plays a role in the efficient binding between PSI and electron donors, thus promoting electron transfer in eukaryotic organisms [51]. PsaH is a transmembrane protein transported to the chloroplast and binds to the reaction center of PSI. In plants, by connecting to PsaL, they ensure the coordinated operation between PSI and the light-harvesting complex [52].

In addition, after our treatments, some enzymes were activated, such as plastoquinol/plastocyanin oxidoreductase (EC:7.1.1.6), which is found in the cytochrome b6f complex and plays a crucial role in photosynthesis, transferring electrons from PSII to PSI [53]. The ferredoxin-nicotinamide adenine dinucleotide phosphate reductase (EC:1.18.1.2), which acts on plant-type ferredoxins [54], was also activated in PSI because of our treatment [42]. It was found that the HA treatment significantly increased the net photosynthetic rate, stomatal conductance, effective quantum yields of PSI and PSII, the PSI and PSII relative photosynthetic electron transfer rates and photochemical quenching.

The H^+^-transporting ATPase (EC:7.1.2.2) enzyme—which is involved in the transport of ions and generates the H^+^ motive force across the plasma membrane necessary to activate most of the ion and metabolite transport [55]—was also induced by the HA treatment. Similar results were detected by Canellas et al. (2013) [21]. The modulation of the ATPF1B enzyme subunit should also be highlighted; F1B is an F-type H^+^ transport ATPase β-subunit gene in ATP synthase, which, together with other genes, is functionally essential for carbon dioxide assimilation [56].

Additional genes were expressed during the carbon-fixation phase, which led to the activation of key enzymes of the Calvin cycle, such as fructose bisphosphatase (EC:3.1.3.11), phosphoglycerate kinase (EC:2.7.2.3), ribulose biphosphate carboxylase/oxygenase (EC:4.1.1.39), and ribose-5-phosphate isomerase (5.3.1.6). In addition, the enzymes of the C4 dicarboxylic acid pathway were also modulated by the HA treatment; these were pyruvate, phosphate dikinase (EC:2.7.9.1), malate dehydrogenase (EC:1.1.1.37), and alanine transaminase (EC:2.6.1.2). Canellas et al. (2013) [21] and Orsi (2014) [57] achieved similar results.

Following the humic acid treatment, transcriptomic studies showed that photosynthesis-related genes, especially chlorophyll synthesis and light energy capture, were upregulated in C4 maize. Conversely, this study found no correlation with the activity of dark-phase enzymes in C4 plants. The latter function, significantly the increase in the activity of the RuBisCO enzyme, was only detected in C3 plants by Zhi et al. (2024) [58], but in the present study, we also confirm the increase in activity of several dark-phase enzymes in C4 plants, which is a new finding. All of these gene-expression changes together can activate the partial processes of photosynthesis at several points, which can ultimately increase the yield of the crop [6,8].

Carletti et al. (2008) [59] also found in their protein-level studies that HA treatment downregulated glycolytic enzymes. As a dominant biochemical process, glycolysis is crucial in healthy plants. Most carbon metabolized in this way is involved in the plant’s active growth, as many essential compounds are synthesized from it, including nucleic acids, amino acids, fatty acids, or even secondary metabolites. In contrast, we found that some genes were also activated in the glycolysis phase of cell respiration processes following the HA treatment, which were the following: alcohol dehydrogenase (EC:1.1.1.1), pyruvate-phosphate dikinase (EC:2.7.9.1), phosphoglycerate kinase (EC:2.7.2.3), and the fructose-bisphosphatase (EC:3.1.3.11) enzyme genes. In addition, many genes were activated in the oxidative phosphorylation phase because of the HA treatment. Three enzymes of the electron transport chain were modulated, including NADH:ubiquinone reductase (EC:7.1.1.2), which has an H^+^-translocating role, inorganic diphosphatase (EC:3.6.1.1), and H+-transporting two-sector ATPase (EC:7.1. 2.2). The other three genes of the biochemical cycle were also induced following the treatment, namely, ND2, a protein subunit of NADH, and the ATPF1B and ATPeF1A subunits of the ATPase.

The oxidative phosphorylation step provides the most energy for maintaining homeostasis, and the activation of this process also affects crop growth [60]. Yang et al. (2023) [61] found that HA treatment positively influenced oxidative phosphorylation processes in maize under stress conditions. Our treatments induced 3–3 ribosomal proteins in the large and small subunits. Roomi et al. (2018) [62] also detected positive changes in the induction of ribosomal proteins due to a humic acid treatment. The activation of enzymes in the glycolytic pathway and the upregulation of ribosomal protein indicated a stimulation of energy metabolism and protein synthesis. Compared to the control, the HA+AA combined treatment similarly upregulated some genes of the light and dark phases of photosynthesis, positively affected the oxidative phosphorylation step in cellular respiration, and stimulated the function of ribosomes.

During the light phase of photosynthesis, the PSII activity increased compared to the control and humic acid treatment. In addition to psbP, psbB, psbJ, and psbY were also induced. PSII is the water-splitting enzyme complex of photosynthesis and consists of many protein subunits. Several studies have shown that PsbP in the OEC concentrates Ca^2+^ ions [63]; additionally, it modulates the level of Cl^−^ required for water oxidation and plays a direct role in the light-induced assembly of the OEC manganese cluster [64]. psbB is an antenna protein that forms the electron transport chain together with psbA and psbD subunits, so PSII does not work in its absence [65,66,67]. Studies suggest that PsbJ plays a role in regulating electron flow and promotes the assembly of the PsbP subunit and PSII [68,69]. Inactivated psbJ tobacco plants were unable to grow photo autotrophically and suffered from light sensitivity [70]. In some studies, psbY has been shown to interact with Cyt b559 directly [71,72,73], which is involved in cyclic electron flow around PSII, thereby protecting PSII from photoinhibition [74,75].

In contrast, the activity of PSI was lower compared to the HA treatment but was activated compared to the control, with the overrepresentation of the psaK gene. The primary function of the small subunits, including psaK, is to stabilize the structures of the antenna system and photosystem I [76]. The cytochrome b6f complex was also activated compared to the control and HA treatments, overrepresenting two genes, petA and petB. The two structural subunits involved in constructing the cytochrome b6f enzyme complex promote the plastoquinol/plastocyanin transformation in the thylakoid membrane of chloroplasts. During photosynthesis, the cytochrome b6f complex mediates the transfer of electrons and energy between PSII and PSI while transferring protons from the chloroplast stroma across the thylakoid membrane into the lumen [77]. Electron transport via cytochrome b6f is responsible for establishing the proton gradient that drives ATP synthesis in chloroplasts [78].

Furthermore, the ATPF1A gene of the ATPase enzyme was activated. In [79], upregulation of this gene was also found during genome-wide analysis in Dahurian larch species under environmental stress. In addition, all of the enzymes already activated in the electron transport chain as a result of the HA treatment and described were overexpressed here as well, caused by the combined treatment. In addition to the enzymes activated due to the HA treatments, some others were also overexpressed in the combined treatments in the dark phase. These are aspartate transaminase (EC:2.6.1.1) and oxaloacetate decarboxylase (EC:4.1.1.3). Aspartate transaminase is involved in the conversion of aspartate to oxaloacetate; oxaloacetate decarboxylase is involved in the conversion of oxaloacetate to pyruvate [80]. Other enzymes stimulated in the HA treatment were not upregulated with the HA+AA treatment. These were EC:5.3.1.6, EC:2.7.2.3, and EC 1.1.1.37 (see their detailed functions above).

The combined HA+AA treatments and their complex effects on photosynthesis’s light and dark phases have been little known until now. Our study also sheds light on the effects of combined treatments on the PSII and PSI photochemical systems. As a result of the HA+AA combined treatment—in addition to the three enzymes catalyzed by HAs (see above)—additional enzymes were activated during the oxidative phosphorylation process, such as NADH-ubiquinone reductase (EC:1.6.5.9), quinol-cytochrome-c reductase (EC:7.1.1.8), and cytochrome-c-oxidase (EC:7.1.1.9). EC:7.1.1.8 has a vital role in the oxidization of the quinol and transfer electrons to cytochromes, playing a unique role in managing the shift from two-electron to one-electron processes within the electron transport chain [81]. EC:1.6.5.9 catalyzes the transfer of electrons from NADH to coenzyme Q10 and helps to translocate protons across the inner mitochondrial membrane [82]. EC:7.1.1.9 is the last enzyme in the electron transport chain. Each of the four cytochrome-c molecules in the mitochondrial system donates an electron to the enzyme, which transfers them to an oxygen molecule and four protons, ultimately forming two water molecules [83]. Some overexpressed genes, in addition to enzymes, include ND4, ndhK, ndhD, and ATPF1A, which contributed to making cellular respiration more efficient. In [84], it was demonstrated that humic acids act as electron mediators to enhance electron transfer in living systems, promoting sustainable energy optimization within the cell.

So far, it has not been described at which specific points HA+AA combined treatments stimulate the cellular respiration processes of C4 plants. In addition to the above, HA+AA combined treatments modulated ribosome subunits’ functioning. Seven genes were activated, of which only one affected the small subunit. The large ribosomal subunit catalyzes the key chemical event in protein synthesis and peptide bond formation. This also shows that protein synthesis was activated due to the combined treatment, which is supported by numerous studies [25,26,31,46].

What is new compared to the control and HA treatment is that the combined treatments resulted in the overexpression of five genes in the process of purine metabolism, including thiamine biosynthesis. Of these, the induction of the enzyme nucleoside-triphosphate phosphatase (EC:3.6.1.15) should be highlighted. The enzyme is capable of breaking the hydrogen bonds of RNA strands found in living systems; thus, it can function as an inhibitor in some attack processes caused by biotic stress. The increase in enzyme activity has a possible inhibitory effect on viral replication [85].

In addition to the positive effects, the application of both HA solutions also caused the suppression of some genes. The decreases in gene expression caused by the HA treatments are not definitive, since only 1-2 genes were overexpressed or downregulated in each biochemical process. However, it should be noted that such processes involved the metabolism of glutathione and methane (2-2 genes). The HA+AA combined treatment further strengthened this effect, as the KEGG analyses showed that three genes involved in glutathione and four in methane metabolism were downregulated. With the combined treatment, four genes were also underexpressed for pyruvate metabolism. These results contradict several studies reporting the stress-relieving effects of HAs. Among the carefully examined data presented in the Section 1, we could not prove a positive effect in the stress-relieving, antioxidant production-enhancing, or yield content-increasing parameters.

## 4. Materials and Methods

### 4.1. Plant Materials

At the Georgikon Campus of the Hungarian University of Agricultural and Life Sciences in Keszthely, Hungary, we cultivated P9718E WAXY hybrid maize plants under field conditions. The area typically has Ramann’s brown forest soil. The plowed layer was 25–30 cm long, with a humus content of 1.5% and a pH of 7.2. From an experimental perspective, we chose the field’s most homogeneous area, where stems were abundant and plant height was even. The corn plants were planted in 12 × 100 m^2^ plots, with a 70 cm row spacing, 20 cm plant spacing, and 5 cm planting depth. The treatments were set up in a non-randomized block design. Of the 12 identically sized plots, 4 were kept as controls, 4 were treated with HA, and 4 were treated with the HA+AA combination.

The samplings for each study represent plant samples collected from 4-4-4 plots, which can be considered replicates of each other. For the measurements of plant physiological parameters, 10-10 whole maize plants were used in 4-4 replicates per treatment.

For the bioinformatics studies, 30–50 mg samples were taken from each well-developed, healthy maize plant in 4-4 replicates per treatment.

The treatments were applied on 6 and 7 October 2022, with the humic acid solution at 5 l/ha and the HA and AA combined solution at a dose of 2.5 l/ha. The HA solution was concentrated at 0.6 m%, and the combined solution was concentrated at 17.5 m% in amino acids, including proline, methionine, glycine, threonine, valine, and lysine, in equal amounts.

### 4.2. Water Saturation Tests

Plants can temporarily lose a certain proportion of their water content without suffering significant damage or dying. However, a permanent drought upsets the plants’ water balance, as their water intake fails to balance their water release. Knowing the value of the current and critical saturation deficit is particularly important from the point of view of irrigation. The current saturation deficit, or the water deficit, represents the difference between the measured water content during the test and the maximum water content (%).

The critical saturation deficit refers to the water deficit that causes irreversible damage to the plant. By regularly measuring the water content of the leaves, we can establish the evolution of the water deficit and calculate the current water deficit from the obtained water content data [86]. The leaves’ water-saturated deficiency (WSD%) indicates how much water is missing from the tissue compared to total saturation. The following relationship allows us to convert the saturation water deficit and relative water content (RWC%) values to on another:

WSD % = 100 − RWC %(1)

The tests were conducted daily from 14 June 2022 to 24 June 2022 and after the second priming from 14 July 2022 to 27 July 2022 in the 7- and 10-leaf states of the maize. Sampling took place in the early morning hours, between 6 and 7 a.m., in 4 repetitions per treatment. Their weight was measured after cutting 12 identical rectangles of approximately 12 cm^2^ in size from the leaf samples. After a two-hour soaking phase, another weight measurement was followed by two hours of drying (105 °C until constant weight). The weight data after drying were also recorded.

### 4.3. SPAD Measurements

During the measurements, we used the SPAD 502 PLUS portable chlorophyll meter manufactured by Konica Minolta. The device calculates the SPAD index from the ratio of the intensity of red (650 nm) and infrared (940 nm) light passing through the leaf, according to the following equation:

SPAD ≈ log10 (T940/T650) = A650 − A940(2)

where T is the light transmission value at given wavelengths; and A is the absorption value at given wavelengths

For most plants, the SPAD value depends linearly on the concentration of chlorophyll in the leaves [87]. The first series of measurements occurred from 14 June 2022 to 22 June 2022, and the second from 14 July 2022 to 27 July 2022. On each measurement day, the SPAD values of the leaves of thirty plants per treatment were recorded.

### 4.4. NIR Spectrometer Analysis

Near-infrared spectroscopy (NIRS) examines the absorption of light energy in the near-infrared range by the molecules of various substances. Changes in the absorbance of particles of the tested sample are converted into numerical values by the system. The measurements were estimated using the FOSS NIRS DS2500 model, which can be used in the 400 and 2500 nm range. The homogeneous samples were poured into the small glass-bottomed vessel belonging to the measuring instrument, leaving no transparent surface. The scanning was performed in 4 repetitions per sample after selecting the appropriate plant species and tissue type (leaf or seed). The machine recorded, stored, and converted the data into an Excel file for further use [88,89]. Whole leaf samples were collected from the plants. The following parameters were examined in three phenophases (milk ripening, wax ripening, and full ripening) in 4 repetitions: protein, fat, crude fiber, ash, and starch content.

In addition, we also performed the exact measurements on samples taken from the grains at harvest in 4 replicates.

### 4.5. Post-Harvest Tests

After harvesting, the following grain parameters were examined in 4 replicates per treatment: weight (g), length (cm), maize tube diameter (mm), number of rows on the tubes, embedment (tube embeddedness), tube weight (g), grain weight (g), maize kernels number/tube, and average grain weight (g).

### 4.6. Statistical Analyses

Data were expressed as the mean ± SD. Differences among the data were assessed statistically by one-way analysis of variance (ANOVA) as sources of variation. A multiple F-test was used to determine significance among the means for significant main effects. The significance level was set at *p* < 0.05, with a confidence level of 95%.

### 4.7. Transcriptomic Analyses

Two days after the second priming in the vegetative phase (10 leaves phase), 30–50 mg of leaves were collected from each treatment. The samples were placed in 1-1 mL RNALater (Invitrogen by Thermo Fisher Scientific Inc., Waltham, MA, USA) solution and stored at −20 °C until further processing. The mRNA was purified from leaf tissue samples containing RIN ≥ 7 total RNA with paramagnetic NEXTFLEX^®^ Poly(A) Beads 2.0 beads and, after fragmentation, strand-specific NGS library preparation was performed using the NEXTFLEX^®^ Rapid Directional RNA-Seq 2.0 kit. The completed pooled libraries were sequenced on the Illumina NovaSeq 6000 genome sequencing platform [90].

Raw reads received from the sequencing company were subjected to quality control, low-quality regions were removed, and the reads were filtered using FastQC (https://timkahlke.github.io/LongRead_tutorials/QC_F.html (accessed 1 August 2024)) and Trimmomatic software; http://www.usadellab.org/cms/index.php?page=trimmomatic (accessed 2 August 2024 [91]. In the next step, a so-called de novo transcript was reconstructed from the prefiltered and qualitatively appropriate short reads without the help of a reference genome using Trinity software; http://TrinityRNASeq.sourceforge.net (accessed 2 August 2024) [92]. The Trinity sequence assembler software can assemble short nucleotide sequences into longer contigs.

All contigs of the de novo transcriptome were identified using the CloudBlast sequence alignment program used by OmicsBox BioBam program packages [93]. GO (gene Ontology) terms were then associated (i.e., mappEd) with Blast2GO software; https://www.blast2go.com/ (accessed 3 August 2024) [94] to blasted sequences. Then, functional annotation was performed using EggNOG-mapper, which is suitable for the functional annotation of new, so far unknown transcriptomes [95].

Estimating the expression level of the de novo transcriptome is a necessary step to perform differential expression analysis. In the absence of a reference sequence, the software creates a map of the transcripts. After the mapping, the software quantifies the reads considering the gene coordinates [96]. The software records a summary of the results in a count table output file.

Pairwise differential expression analysis was performed using the NOIseq software; https://bioconductor.org/packages/release/bioc/html/NOISeq.html (accessed 3 August 2024) package between the contigs of the control and treated samples to determine which genes were expressed to a statistically significantly different (probability > 0.9) extent as a result of each treatment [97,98].

With the help of the test, compared with the data of the de novo blasted mapped annotated transcriptome, among the down- and upregulated sequences from the treated herds, we selected those that were under- or overrepresented, that is, those with a presence much lower or greater than the average of the control herds. We grouped them based on their molecular functions, and with their help, we could better understand the effects of our treatments on biological functions [99,100].

Combined pathway analysis was used to identify the biochemical pathways and biological mechanisms that changed as a result of the treatments using the following two public databases: Plant Reactome [101] and KEGG [102].

## 5. Conclusions

We primed corn plants with HA or HA and AA combined treatments in the present study. We aimed to investigate the contradictory effects revealed by field experiments in the preliminary literature sources and with bioinformatics methods. Our study confirms that humic-acid-containing organic materials have a beneficial impact on particular plant physiological parameters in corn, whose effects synergize the amino acid combination. Our genome-wide transcriptomic studies reveal deeper connections, according to which humic acid and combined primings significantly impact photosynthetic and cellular respiration processes and the functioning of ribosomes. In addition, we could not confirm any of the additional beneficial impacts described in some studies in the literature (stress-relieving ability, hormone-mediated effect, antioxidant effects, etc.). In summary, it can be said that, given all these results, the use of humic acid products (and their combinations) is also acceptable on organic farms, which are gaining prominence today, as they can be of excellent use in environmentally friendly farming and support sustainable, regenerative agriculture.

## Figures and Tables

**Table 1:** NIR measurements in maize leaves.

Treatment/Parameter	DM	Protein	Ash	ADF	NDF
**Control**	21.46 ^c^ ± 0.25	3.81 ^c^ ± 0.097	0.519 ^c^ ± 0.052	4.72 ^b^ ± 0.059	12.5 ^c^ ± 0.15
**HA**	22.59 ^b^ ± 0.33	4.06 ^bc^ ± 0.096	0.65 ^b^ ± 0.051	4.94 ^a^ ± 0.056	13.042 ^b^ ± 0.19
**HA+AA**	23.81 ^a^ ± 0.29	4.25 ^a^ ± 0.99	0.959 ^a^ ± 0.041	4.92 ^a^ ± 0.05	13.72 ^a^ ± 0.23

The mean and standard deviation (SD) are represented, and the small letters above the mean (^a, b, c^) indicate statistically significant differences between variables (i.e., treatments).

**Table 2:** Post-harvest NIR measurements of the maize grain.

Treatment	Parameters (Harvest)					
	Protein	Moisture	Fat	Crude Fiber	ASH	Starch
**Control**	10.49 ^a^ ± 0.17	19.88 ^a^ ± 0.03	3.34 ^c^ ± 0.01	2.51 ^b^ ± 0.03	1.14 ^b^ ± 0.01	56.61 ^b^ ± 0.56
**HA**	9.59 ^b^ ± 0.22	18.63 ^b^ ± 0.34	3.37 ^b^ ± 0.01	2.13 ^c^ ± 0.05	1.20 ^a^ ± 0.03	59.34 ^a^ ± 0.32
**HA+AA**	10.37 ^a^ ± 0.08	19.86 ^a^ ± 0.04	3.43 ^a^ ± 0.01	2.60 ^a^ ± 0.05	1.13 ^b^ ± 0.01	54.39 ^c^ ± 0.51

The mean and standard deviation (SD) are represented. The small letters above the mean (^a, b, c^) indicate statistically significant differences between variables (i.e., treatments).

**Table 3:** Yield parameters of maize plants.

Treatment	Control		HA		HA+AA	
Yield Parameter	Mean	SD	Mean	SD	Mean	SD
**Weight (g)**	136.875 ^c^	15.73	194.965 ^b^	13.63	220.61 ^a^	18.93
**Length (cm)**	17.6 ^c^	0.54	18.855 ^ab^	0.60	19.545 ^a^	0.56
**Diameter (mm)**	41.00 ^c^	1.52	42.95 ^b^	1.73	44.65 ^a^	2.16
**Number of Lines**	16.00	1.59	15.20	1.20	15.20	1.20
**Serial Number per Seed**	31.00 ^c^	1.56	34.80 ^b^	1.61	36.20 ^a^	2.84
**Embedding (Occupancy)**	7.35 ^a^	1.46	4.35 ^b^	1.90	3.50 ^c^	1.99
**Cob Weight (g)**	24.00 ^c^	3.01	32.06 ^b^	1.97	40.08 ^a^	2.55
**Grain Weight (g)**	116.52 ^c^	11.02	156.6 ^b^	11.15	189.52 ^a^	17.31
**Number of Maize Kernels**	269.45 ^c^	72.34	379.00 ^b^	79.96	428.30 ^a^	111.71

The mean and standard deviation (SD) are represented. The small letters after the averages (^a, b, c^) indicate statistically significant differences between the variables (i.e., treatments) in a row.

## Data Availability

NGS Library Preparation and Sequencing: After filtering and quality control, an average of 22–25 million bp single-end reads per sample, which was 63 bp in length, were obtained from the NGS. Raw reads were deposited in the National Center for Biotechnology Information (NCBI) database under the following accessions: (1) Repository name: Maize treated by humic acid content plant conditioner; Data identification number: PRJNA1141959; Direct URL to data: https://www.ncbi.nlm.nih.gov/bioproject/PRJNA1141959 (accessed 30 July 2024). (2) Repository name: Maize_control_R1; Data identification number: SRR30037659; Direct URL to data: https://www.ncbi.nlm.nih.gov/sra/?term=SRR30037659 (accessed 30 July 2024). (3) Repository name: Maize_humic acid_R1; Data identification number: SRR30037658; Direct URL to data: https://www.ncbi.nlm.nih.gov/sra/?term=SRR30037658 (accessed 30 July 2024). (4) Repository name: Maize_humic acid_amino acid_R1; Data identification number: SRR30037655; Direct URL to data: https://www.ncbi.nlm.nih.gov/sra/?term=SRR30037655 (accessed 30 July 2024). Preprocessing and RNA-seq de novo assembly: After pre-screening, a de novo transcriptome was generated using 675,694 short reads. The short reads were derived from the bulked biological samples sequenced of four replicates per treatment. The de novo transcriptome reconstruction resulted in 2279 transcripts, from which 1980 identified genes were determined. The super transcriptome is available in the NCBI public database under the following accession number: https://submit.ncbi.nlm.nih.gov/subs/tsa/SUB14644426/overview (accessed 30 July 2024).

## Supplementary Materials

The following supporting information can be downloaded at: https://www.mdpi.com/article/10.3390/ijms252413280/s1.
